# Supplementary material for: The miRNA bantam regulates growth and tumorigenesis by repressing the cell cycle regulator tribbles
Source: Life Sci Alliance. 2019 Jul 22;2(4):e201900381. doi: 10.26508/lsa.201900381 (PMC6653758; doi:10.26508/lsa.201900381)
Supplement: Supplementary file 1 [file LSA-2019-00381_Supplemental_Data_1.docx]

**List of Genotypes**

Genotypes – Figure 1:

(B) ptc-Gal4/+; trbl-GFP/+

(C) ptc-Gal4/+; trbl-GFP/UAS-bantam-A

(D) ptc-Gal4/+; tub-trbl-sensor/+

(E) ptc-Gal4/+; tub-trbl-sensor/UAS-bantam-A

(F) ptc-Gal4/+; tub-trbl-mut-sensor/+

(G) ptc-Gal4/+; tub-trbl-mut-sensor/UAS-bantam-A

(H) hs-Flp122/+; tub-trbl-sensor/+; FRT80, bantam-delta1/FRT80, M, arm-lacZ

(I) hs-Flp122/+; tub-trbl-mut-sensor/+; FRT80, bantam-delta1/FRT80, M, arm-lacZ

(J) hs-Flp/+; tub-trbl-sensor/+; FRT82b, wts-x1/FRT82b, RFP

(K) hs-Flp/+; tub-trbl-sensor/+; FRT82b, wts-x1/FRT82b, RFP

Genotypes – Figure 2:

(B) ap-Gal4, UAS-fly-FUCCI/+; tub-Gal80-ts/UAS-lacZ

(C) ap-Gal4, UAS-fly-FUCCI /+; tub-Gal80-ts/UAS-trbl

(D) ap-Gal4, UAS-fly-FUCCI /+; tub-Gal80-ts/UAS-trbl-RNAi

(E) ap-Gal4, UAS-GFP/+; tub-Gal80-ts/UAS-lacZ

(F) ap-Gal4, UAS-GFP/+; tub-Gal80-ts/UAS-trbl

(H-1) MS1096-Gal4/+; UAS-GFP/+

(H-2) MS1096-Gal4/+; UAS-trbl-RNAi/+

(H-3) MS1096-Gal4/+; UAS-trbl/+

(J-1) MS1096-Gal4/+; UAS-GFP/+

(J-2) MS1096-Gal4/+; UAS-trbl-RNAi/+

(J-3) MS1096-Gal4/+; UAS-trbl/+

Genotypes – Figure 3:

(A) ap-Gal4, UAS-GFP/+; tub-Gal80-ts/UAS-lacZ

(B) ap-Gal4, UAS-GFP/+; tub-Gal80-ts/UAS-trbl

(C) ap-Gal4, UAS-GFP/+; tub-Gal80-ts/UAS-bantam-D

(D) ap-Gal4, UAS-GFP/+; tub-Gal80-ts/UAS-bantam-D, UAS-trbl

(E) ap-Gal4, UAS-GFP/+; tub-Gal80-ts, UAS-yki/UAS-lacZ

(F) ap-Gal4, UAS-GFP/+; tub-Gal80-ts, UAS-yki/UAS-trbl

Genotypes – Figure 4:

(A) ap-Gal4, UAS-GFP/+; tub-Gal80-ts/UAS-lacZ

(B) ap-Gal4, UAS-GFP/+; tub-Gal80-ts/UAS-trbl-RNAi

(C) ap-Gal4, UAS-GFP/+; tub-Gal80-ts/UAS-hid-RNAi

(D) ap-Gal4, UAS-GFP/+; tub-Gal80-ts/UAS-hid-RNAi, UAS-trbl-RNAi

(F) ap-Gal4, UAS-GFP/+; tub-Gal80-ts/UAS-lacZ

(G) ap-Gal4, UAS-GFP/+; tub-Gal80-ts/UAS-trbl-RNAi

(I) ap-Gal4, UAS-GFP/+; UAS-bantam-sponge-RFP/UAS-lacZ

(J) ap-Gal4/+; UAS-bantam-sponge-RFP/UAS-hid-RNAi, UAS-trbl-RNAi

Genotypes – Figure 5:

(A) ap-Gal4, UAS-GFP/+; tub-Gal80-ts, UAS-EGFR/+

(B) ap-Gal4, UAS-GFP/+; tub-Gal80-ts, UAS-EGFR/UAS-bantam-D

(C) ap-Gal4, UAS-GFP/+; tub-Gal80-ts, UAS-EGFR/UAS-bantam-D, UAS-trbl

(D) ap-Gal4, UAS-GFP/+; tub-Gal80-ts, UAS-EGFR/UAS-trbl-RNAi

(E) ap-Gal4, UAS-GFP/UAS-Myt1-RNAi; tub-Gal80-ts, UAS-EGFR/+

(F) ap-Gal4, UAS-GFP/UAS-stg; tub-Gal80-ts, UAS-EGFR/+

(H) UAS-pnut-RNAi/+; ap-Gal4, UAS-GFP/+; tub-Gal80-ts/+

(I) ap-Gal4, UAS-GFP/+; tub-Gal80-ts/UAS-trbl-RNAi

(J) ap-Gal4, UAS-GFP/UAS-stg; tub-Gal80-ts/+

(K) ap-Gal4, UAS-GFP, UAS-p35/+; tub-Gal80-ts/+

(L) UAS-pnut-RNAi/+; ap-Gal4, UAS-GFP, UAS-p35/+; tub-Gal80-ts/UAS-trbl-RNAi

(M) UAS-pnut-RNAi/+; ap-Gal4, UAS-GFP, UAS-p35/UAS-stg; tub-Gal80-ts/+

Genotypes – Supp. Figure 1:

(A) trbl-GFP/bantam-lacZ

(B) ptc-Gal4/UAS-GFP

(C) ptc-Gal4/+; trbl-GFP/UAS-bantam-EP

(D) ptc-Gal4/+; tub-trbl-sensor/UAS-bantam-EP

(E) ptc-Gal4/+; tub-trbl-mut-sensor/UAS-bantam-EP

(F-1) trbl-GFP/+

(F-2) trbl-GFP, UAS-bantam-EP/+

(F-3) trbl-GFP, UAS-bantam-EP/bantam-delta-1

Genotypes – Supp. Figure 2:

(A) hs-Flp122/+; tub-trbl-sensor/+; FRT80/FRT80, M, arm-lacZ

Genotypes – Supp. Figure 3:

(A) ptc-Gal4/+; trbl-GFP/+

(B) ptc-Gal4/+; trbl-GFP/UAS-trbl-RNAi

Genotypes – Supp. Figure 4:

(A-1) nub-Gal4, UAS-GFP/+

(A-2) nub-Gal4/+; UAS-trbl-RNAi/+

(A-3) nub-Gal4/+; UAS-trbl/+

(B-1) nub-Gal4, UAS-GFP/+

(B-2) nub-Gal4/+; UAS-trbl-RNAi/+

(B-3) nub-Gal4/+; UAS-trbl/+

Genotypes – Supp. Figure 5:

(A) ap-Gal4, UAS-GFP/+; tub-Gal80-ts/UAS-hid-RNAi, UAS-trbl-RNAi

(B) ap-Gal4, UAS-GFP/+; tub-Gal80-ts/UAS-lacZ

(C) ap-Gal4, UAS-GFP/+; tub-Gal80-ts/UAS-trbl-RNAi

(D) ap-Gal4, UAS-GFP, UAS-p35/+; tub-Gal80-ts/UAS-trbl-RNAi

(E) ap-Gal4, UAS-GFP/UAS-miR-RHG; tub-Gal80-ts/+

(F) ap-Gal4, UAS-GFP, UAS-p35/+; tub-Gal80-ts/UAS-trbl-RNAi

(G) ap-Gal4, UAS-GFP, UAS-p35/+; tub-Gal80-ts/UAS-trbl-RNAi

(H) ap-Gal4, UAS-GFP/UAS-trbl-RNAi-KK; tub-Gal80-ts/+

(I) ap-Gal4, UAS-GFP/UAS-trbl-RNAi-KK; tub-Gal80-ts/UAS-hid-RNAi

(J) ap-Gal4, UAS-GFP, UAS-p35/UAS-trbl-RNAi-KK; tub-Gal80-ts/+

Genotypes – Supp. Figure 6:

(A) ap-Gal4, UAS-GFP/+; tub-Gal80-ts/UAS-lacZ

(B) ap-Gal4, UAS-GFP/UAS-stg; tub-Gal80-ts/+

(C) ap-Gal4, UAS-GFP/+; tub-Gal80-ts/UAS-lacZ

(D) ap-Gal4, UAS-GFP/UAS-stg; tub-Gal80-ts/+

(E) ap-Gal4, UAS-GFP, UAS-p35/UAS-stg; tub-Gal80-ts/+

Genotypes – Supp. Figure 7:

(A) ap-Gal4, UAS-GFP/+; tub-Gal80-ts/UAS-lacZ

(B) ap-Gal4, UAS-GFP/UAS-Myt1-RNAi; tub-Gal80-ts/+

(C) ap-Gal4, UAS-GFP/UAS-stg; tub-Gal80-ts/+

(D) ap-Gal4, UAS-fly-FUCCI/+; tub-Gal80-ts/UAS-lacZ

(E) ap-Gal4, UAS-fly-FUCCI/UAS-Myt1-RNAi; tub-Gal80-ts/+

(F) ap-Gal4, UAS-fly-FUCCI/UAS-stg; tub-Gal80-ts/+

**Supplementary References**

Vissers, J.H., Manning, S.A., Kulkarni, A., and Harvey, K.F. (2016). A Drosophila RNAi library modulates Hippo pathway-dependent tissue growth. Nat Commun 7, 10368.
